# Cortical effects of wrist tendon vibration during an arm tracking task in chronic stroke survivors: An EEG study

**DOI:** 10.1371/journal.pone.0266586

**Published:** 2023-12-21

**Authors:** Dylan B. Snyder, Scott A. Beardsley, Allison S. Hyngstrom, Brian D. Schmit

**Affiliations:** 1 Department of Biomedical Engineering, Marquette University and Medical College of Wisconsin, Milwaukee, Wisconsin, United States of America; 2 Department of Physical Therapy, Marquette University, Milwaukee, Wisconsin, United States of America; West Virginia University, UNITED STATES

## Abstract

The purpose of this study was to characterize changes in cortical activity and connectivity in stroke survivors when vibration is applied to the wrist flexor tendons during a visuomotor tracking task. Data were collected from 10 chronic stroke participants and 10 neurologically-intact controls while tracking a target through a figure-8 pattern in the horizontal plane. Electroencephalography (EEG) was used to measure cortical activity (beta band desynchronization) and connectivity (beta band task-based coherence) with movement kinematics and performance error also being recorded during the task. All participants came into our lab on two separate days and performed three blocks (16 trials each, 48 total trials) of tracking, with the middle block including vibration or sham applied at the wrist flexor tendons. The order of the sessions (Vibe vs. Sham) was counterbalanced across participants to prevent ordering effects. During the Sham session, cortical activity increased as the tracking task progressed (over blocks). This effect was reduced when vibration was applied to controls. In contrast, vibration increased cortical activity during the vibration period in participants with stroke. Cortical connectivity increased during vibration, with larger effect sizes in participants with stroke. Changes in tracking performance, standard deviation of hand speed, were observed in both control and stroke groups. Overall, EEG measures of brain activity and connectivity provided insight into effects of vibration on brain control of a visuomotor task. The increases in cortical activity and connectivity with vibration improved patterns of activity in people with stroke. These findings suggest that reactivation of normal cortical networks via tendon vibration may be useful during physical rehabilitation of stroke patients.

## 1. Introduction

Stroke is a major contributor to serious physical long-term disabilities [1] with around 50% of stroke survivors being unable to return to work [2]. Motor dysfunctions such as abnormal muscle synergy patterns, spasticity and paresis [3–5] as well as sensory dysfunctions including deficits in tactile, proprioceptive, pressure and thermal sense are common after stroke [6]. These motor and sensory deficits ultimately result in a functionally corrupt motor control system that has trouble initiating and stopping movements, with the movements that are produced being uncoordinated, slower and less smooth when compared to the neurologically-intact population [7–10]. Stroke patients with both sensory and motor deficits exhibit lower functional outcomes than those with motor deficits alone [11], and sensory impairment is a strong predictor of length of recovery and long-term functional outcomes of stroke survivors [12]. This suggests sensory information plays an important role in motor control and has generated interest in sensory interventions to facilitate stroke rehabilitation.

Supplemental sensory feedback can alter the control of movement of the limb in many ways. The application of an extraneous vibration to neurologically-intact study participants improves motor learning and motor control [13–18]. The application of extraneous vibration or somatosensory electrical stimulation to the limb of people with stroke improves spasticity, balance control, hand function, and arm motor control [14–16, 18–23]. Previously, Conrad and colleagues (2011) demonstrated that applying vibration to the wrist flexor tendons of stroke survivors improves performance during a figure-8 tracking task [14]. Tendon vibration in stroke survivors also improves hand end-point stability for targeted arm movements and within unstable workspaces [15, 16].

While tendon vibration improves tracking performance and end-point stabilization in stroke survivors, the cortical mechanism/s underlying the improvements are unclear. A study examining the effect of wrist flexor tendon vibration on spinal cord stretch reflex activity following stroke found no modulation of the biceps or triceps stretch reflexes during vibration, suggesting that vibration-induced improvements in tracking performance and end-point stabilization may arise from nervous system changes at the supraspinal level [24]. A transcranial magnetic stimulation (TMS) study found that vibration at the muscle can modulate the excitability of the motor cortical circuits and increase motor evoked potentials [25], furthering the idea that vibration induces supraspinal changes during motor control. Understanding the mechanism(s) behind these improvements would facilitate and enhance current stroke rehabilitation therapies.

Vibration may induce widespread changes in cortical networks. When vibration is applied to wrist flexor tendons during a motor task, improvements in motor function are not isolated to the wrist but are seen throughout the arm [14–16]. This observation suggests that vibration enhances not only neural structures linked to the stimulated area, but also areas not directly associated with stimulation, possibly by way of altered cortical networks [14–16]. This concept is further supported by TMS and transcranial direct current stimulation (tDCS) studies that show increased excitability of the cortex in regions distant from the locus directly associated with the sensory stimulus [26–28]. The possibility that an enhanced sensory signal may excite widespread cortical networks raises prospects for using these modalities for rehabilitation in stroke.

Electroencephalography (EEG) can be used to gain a deeper insight into the motor control system. Analysis of EEG has led to the discovery of beta band (12-30Hz) power fluctuations above the sensorimotor cortex that are fundamental for motor control [29, 30]. Event related desynchronization (ERD), a decrease in power from rest, within the beta band is generally thought to indicate cortical activation [29–31]. In stroke survivors with motor and sensory deficits, EEG has revealed decreased activity in the sensorimotor cortex [32–35] and decreases in functional connectivity throughout the brain, particularly in the lesioned hemisphere [36–39]. The deficits in stroke motor control may arise from the sensorimotor cortex’s inability to generate appropriate motor commands and/or to correctly process sensory feedback [32–39]. Changes in stroke EEG network activity/connectivity relate to functional/behavioral outcomes, and indicate that brain networks normalize with recovery [32, 40–43].

The purpose of this study was to determine the cortical effects of wrist flexor tendon vibration during visuomotor tracking in people with stroke. Tendon vibration is thought to increase proprioceptive sensory information by activating Ia-afferent neurons [44, 45]. The flow of additional proprioceptive information to the cortex via tendon vibration might boost task-relevant proprioceptive signals of the limb through a stochastic resonance process, and help the system overcome the sensory deficits typically seen in people with stroke [46]. We hypothesized that application of tendon vibration to wrist flexor tendons causes increased cortical activity and connectivity in the regions displaying cortical deficits after stroke.

## 2. Materials and methods

### 2.1. Participant population

A sample of 10 chronic stroke participants and 10 age-matched neurologically-intact controls participated in this study. Means and standard deviations for the groups were reported throughout the text. Stroke participants (5 female, aged 61.2±10.9yr) were required to be at least 1-year post stroke and experience upper extremity hemiparesis. Stroke participants were excluded if they had diagnoses of any other neurological disorder or recent treatment that interfered with neuromuscular function, such as botulinum toxin injection. Stroke participants completed the experiment using their paretic arm, whereas controls used their non-dominant arm. The impairment level of stroke participants was assessed using the upper extremity Fugl-Meyer Assessment (FMA) which consists of a motor portion (maximum score 66) and sensory/proprioception portion (maximum score 12) [47], and the Semmes-Weinstein monofilament test for sensation [48]. The FMA examines reflex activity, strength, and proprioception, among others, and often compares the unaffected side to the affected side to determine the level of stroke impairment (lower values indicate larger impairments). The monofilament test consists of fibers that require varying amounts of force to bend. The fibers were cycled through and applied to participants until they could no longer detect the applied pressure. The lowest level of sensation (grams force) participants could detect was recorded. The monofilament test was performed at seven locations on the palmar surface of the paretic hand and averaged (distal phalanx of the small finger, index finger and thumb; proximal phalanx of the small and index finger; thenar and hypothenar). Control participants (4 female, aged 60.7±11.6yr) reported no history of stroke or any other upper extremity pathology. Detailed demographic data for all participants is shown in Table 1. All participants gave written informed consent, and all procedures were approved by the Marquette University Institutional Review Board in accordance with the Declaration of Helsinki.

**Table 1:** Demographic and clinical data for stroke and control participants.

Participant Identifier	Sex	Age (yr)	Time after Stroke (yr)	Arm Tested	Fugl-Meyer (Motor:66)	Fugl-Meyer (Sensory:12)	Monofilament (g,sensation)
S1	F	60	23	R	63	12	0.08 (-)
S2	F	79	7	R	62	11	0.15 (-)
S3	F	67	30	L	29	12	0.05 (N)
S4	M	64	16	L	61	8	0.19 (-)
S5	F	66	26	R	51	4	60.00 (---)
S6	M	61	11	R	31	12	0.35 (-)
S7	F	65	13	L	38	8	94.29 (---)
S8	M	59	14	L	23	8	60.00 (---)
S9	M	36	8	L	21	8	71.43 (---)
S10	M	55	15	R	27	12	37.43 (---)
C1	F	68	-	L	-	-	-
C2	M	61	-	L	-	-	-
C3	M	51	-	L	-	-	-
C4	F	77	-	L	-	-	-
C5	F	57	-	L	-	-	-
C6	M	67	-	L	-	-	-
C7	M	65	-	L	-	-	-
C8	F	63	-	L	-	-	-
C9	M	34	-	R	-	-	-
C10	M	64	-	L	-	-	-

Stroke (S) and control (C) participants. The “Arm Tested” corresponded to the paretic side for stroke participants and the non-dominant hand for control participants. “Fugl-Meyer” indicates the Fugl-Meyer upper extremity score (Motor: maximum of 66; Sensory: maximum of 12). Monofilament values indicate the average force in grams (g) across seven hand locations tested with the degree of sensation (N: normal (g≤0.07), ‘-’: diminished light tough (0.07>g≤0.4), ‘--’: diminished protective sensation (0.4>g≤2.0), ‘---’: loss of protective sensation (2.0>g≤180.0), ‘----’: deep pressure sensation only (g>180)). (F: female; M: male; R: right; L: left).

### 2.2. Physiological measurements

A 64-channel active electrode actiCAP (Brain Products GmbH, Munich, Germany) system was used to record EEG data. EEG electrodes were arranged in the conventional 10–20 system with the reference at FCz and the ground at AFz. The EEG cap was placed on the participant’s head such that the Cz electrode was in line with the prearticular points in the frontal plane and with the nasion and inion points in the sagittal plane. SuperVisc gel (Brain Products GmbH, Munich, Germany) was applied between the scalp and electrodes to lower the electrode impedances below 10kOhms prior to data collection. EEG data were amplified, sampled at 1kHz, filtered from 0.1 to 200Hz and notch filtered at 60Hz using a Synamps^2^ amplifier system (Neuroscan, Charlotte, North Carolina), and recorded using the Neuroscan software, Scan 4.5.

### 2.3. Test apparatus

**Fig 1:**
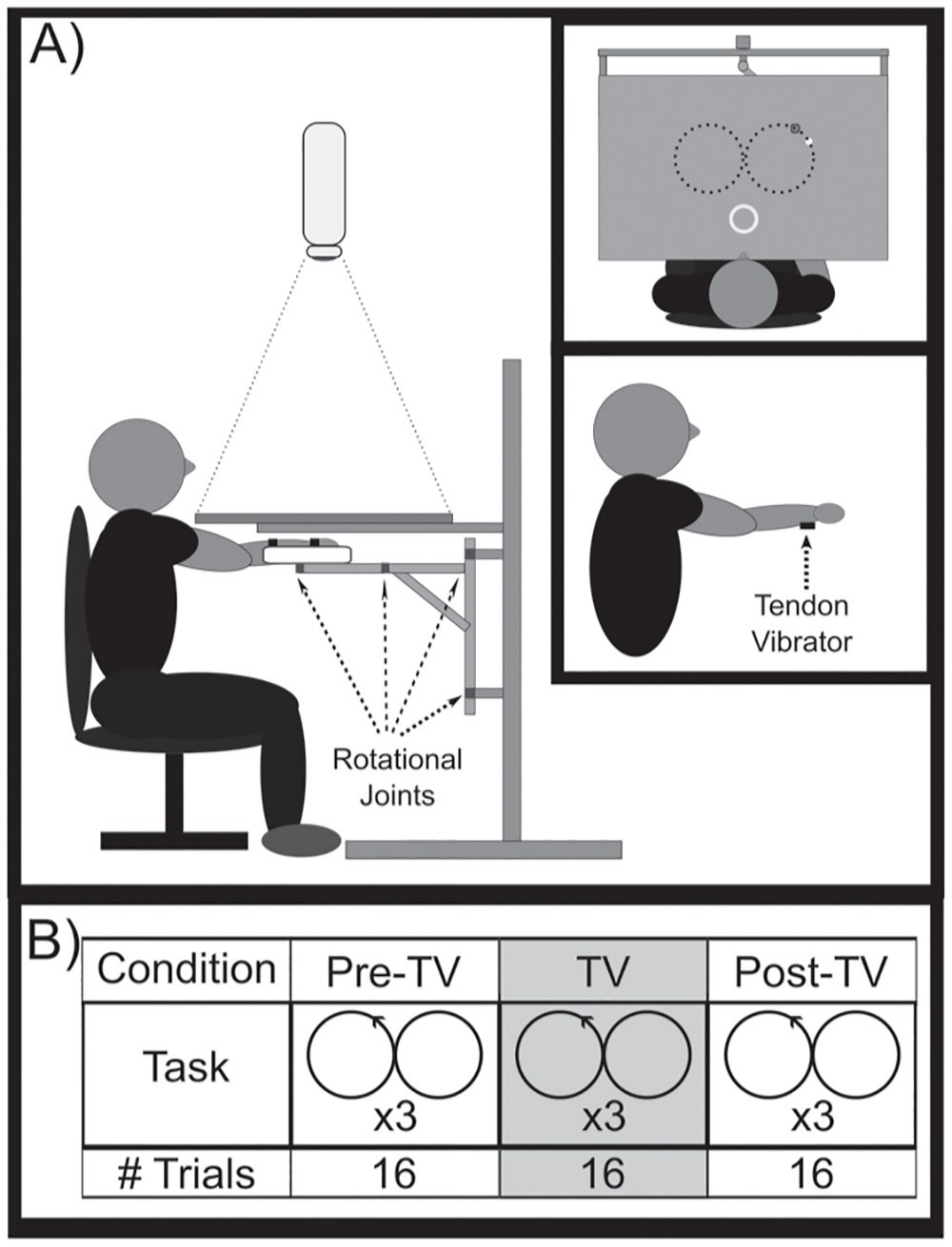
Experimental setup and protocol. A) Illustration of the mechanical linkage and experimental setup from the side (top inset displays the scene from above, bottom inset shows the location of the tendon vibrator). The white cursor projected onto a horizontal screen was linked to hand position. Participants were required to move the cursor from the home location (light gray annulus) to the target (dark gray annulus) and track the target while it moved in a figure-8 pattern. B) Experimental protocol: a single trial consisted of 3 repetitions of the figure-8 pattern. Participants performed three blocks of 16 trials each where the middle block included tendon vibration applied to the wrist flexor tendons.

The study was conducted using a custom-built mechanical linkage (Fig 1A). The linkage constrained movement to the horizontal plane and provided measurements of end-point trajectory using optical encoders (Celesco Transducer Products, Inc., Chatsworth, California; BEI Sensors, Goleta, California) located at each joint. The device frame was constructed using 2.5x2.5cm extruded aluminum (80/20 Inc., Columbia City, Indiana) and contained three rotational joints to allow unrestricted movement in the horizontal plane. While seated at the device, the participant’s forearm was secured via Velcro straps to an Ultra High Molecular Weight Polyethylene tray located at the end of the manipulandum. An overhead projector (NEC Corporation, Tokyo, Japan; model #: NP110, lamp #: NP13LP) displayed hand position and target location (30fps) on an opaque screen (80x60 cm, 9.8 pixels/cm) directly above the plane of hand motion. The device was interfaced with LabVIEW (National Instruments Corporation, Austin, Texas) in order to control the projector display, record (1 kHz sampling rate) kinematic data, and generate digital pulses used to synchronize the timing of movement and EEG data collection.

A custom-made tendon vibrator was affixed to the skin adjacent to the forearm flexor tendons (flexor carpi radialis tendon and flexor carpi ulnaris tendon), distal to the forearm flexor muscles’ bellies and proximal to the wrist (Fig 1A). As these forearm flexor tendons act to flex the wrist, they will be referred to as wrist flexor tendons throughout the text. The vibrator consisted of an offset mass that rotated about the shaft of a motor (Faulhaber Group, Clearwater, FL) and was enclosed in a Teflon casing. The vibrator was then encased by a thin aluminum foil sheet that was electrically grounded to minimize the effect of electromagnetic noise from the vibrator on the EEG recordings. Vibration was applied at 70Hz (1.54N vibratory force) to the wrist flexor tendons of the arm being tested. The 70Hz vibration frequency was selected because it lies within the range of frequencies shown to activate the most muscle spindles at the highest response rate [45].

### 2.4. Experimental protocol

We collected EEG and arm kinematic data while chronic stroke and control participants tracked a target moving in a figure-8 pattern. Vibration was applied to the wrist flexor tendons during a portion of the trials. EEG beta band (13–26 Hz) power fluctuations were used as indicators of brain activity associated with motor function [30, 31, 49]. A novel metric, spatially correlated coherence (SCORCH) of EEG was introduced and used as a measure of functional connectivity between cortical areas.

During the study, a mechanical linkage was used to characterize the participant’s performance on 16 trials of a target tracking task (Fig 1B). Each trial consisted of a baseline period (11.5±1.5s before target presentation), reach period (0–1.75s after target presentation), figure-8 tracking period (1.75–43.75s after target presentation), and return period (~2s between the tracking and baseline periods). Prior to each trial, participants were required to bring a white cursor (r = 0.5cm), linked to horizontal hand position, to the home location (gray annulus, r = 4cm) located ~20 cm in front of the participant. The home location then disappeared, and participants relaxed until the target (red annulus, r = 0.75cm) was presented ~24cm away from the participant on an imaginary line orthogonal to the participant’s chest. Participants then moved their hand as quickly and accurately as possible to the target, at which point the figure-8 tracking period began. During the figure-8 tracking period, the target moved in a figure-8 pattern formed by 2 virtual side-by-side circles (radius = 7cm) centered at the original point of target presentation. As the target moved (0.91rad/s), participants were instructed to follow the target, attempting to keep the cursor in the center of the target. The target moved through the figure-8 pattern three times in a row with the start direction of the figure-8 moving to the left (clockwise or counterclockwise) randomly chosen for each trial.

Before testing, participants completed 8 trials of the tracking task in order to practice the task. During testing, the tracking task was completed in 3 blocks with 16 trials in each block. Tendon vibration (TV) was applied to the middle block of trials allowing for comparisons before (Pre-TV), during (TV), and after (Post-TV) vibration. During the TV block, the vibrator was turned on at the presentation of the target and turned off after the tracking period ended (~44s each trial). The vibrator was not attached to the participants during the Pre-TV and Post-TV blocks. Participants were given breaks halfway through each block and between each block to minimize fatigue.

All participants returned for a control (Sham) session on a separate day with an average of 5 days between sessions. During the control session, the tracking experiment was repeated using the previously described protocol. However, during the session a “sham” vibration was applied in place of the true vibration during the TV block of trials. During the sham vibration trials, the vibrator was placed on the wrist flexor tendons, but the vibrator was not turned on. Vibrator placement was noted and controlled between sessions. The purpose of the Sham session was to assess changes in visuomotor task performance with repetition of the task. The order of the sessions (Vibe vs. Sham) was counterbalanced across participants to prevent ordering effects (1^st^ 5 participants to be scheduled within each group performed the vibration session 1^st^). Please see S1 Text for more information on the experimental protocol.

### 2.5. Data analysis

Trial epochs of the kinematic variables (defined below) and EEG data were extracted from the data (-5 to 42s relative to the start of the figure-8 tracking period, 16 trial epochs per block of trials). From these trial epochs, 6 circle epochs (-0.5 to 7s), approximately representing half of a figure-8 (6.905s), were extracted for each trial (42s). Unique, non-overlapping baseline segments extracted from the -5 to -2s time period at the beginning of the trial epoch were inserted into the -0.5 to 0s time range of the circle epochs while the remaining 7s contained circle tracking data. This process resulted in 96 circle epochs per block of trials used in the subsequent analysis.

#### 2.5.1. Movement kinematics

Hand path kinematic data were processed and analyzed using custom MATLAB scripts (version 2014a, MathWorks, Natick, Massachusetts). Absolute error was calculated as the Euclidean distance of the cursor (hand) from the target,

$$AE\left(t\right)=\sqrt{{\left(C_{X}\left(t\right)-T_{X}\left(t\right)\right)}^{2}+{\left(C_{Y}\left(t\right)-T_{Y}\left(t\right)\right)}^{2}}$$ (1)

Where *AE*(*t*) represents the absolute error, *C*_*X*_(*t*) represents the cursor’s *X* position on the screen, *C*_*Y*_(*t*) represents the cursor’s *Y* position on the screen, *T*_*X*_(*t*) represents the target’s *X* position on the screen, *T*_*Y*_(*t*) represents the target’s *Y* position on the screen, and *t* represents time. Speed was calculated from the x and y cursor (hand) positions obtained from the optical encoders. Absolute error and speed were then epoched resulting in 96 epochs per block. Bad epochs were removed manually by using FieldTrip’s visual inspection code (epochs were removed if the speed variance/kurtosis >2 standard deviations from the mean, ft_rejectvisual, average number removed, 41.9). Standard deviation (SD) of hand speed was calculated for each epoch’s tracking period (0–7s). The MATLAB ‘trapz’ function was used to find the area under the speed curve indicating the total path length of the hand during the tracking period of the epochs. To evaluate motor planning time, the number of sub-movements made by the hand during the epoch tracking period were counted. Sub-movements were identified by local minima in the speed traces with a single sub-movement being considered the activity occurring between consecutive local minima. Hand absolute error and speed were each averaged during the tracking period. All kinematic variables (Hand absolute error, speed, standard deviation (SD) of speed, total path length and number of sub-movements) were averaged across epochs for each participant and compared across conditions to determine if vibration improved visuomotor tracking performance.

#### 2.5.2. EEG processing

**Fig 2:**
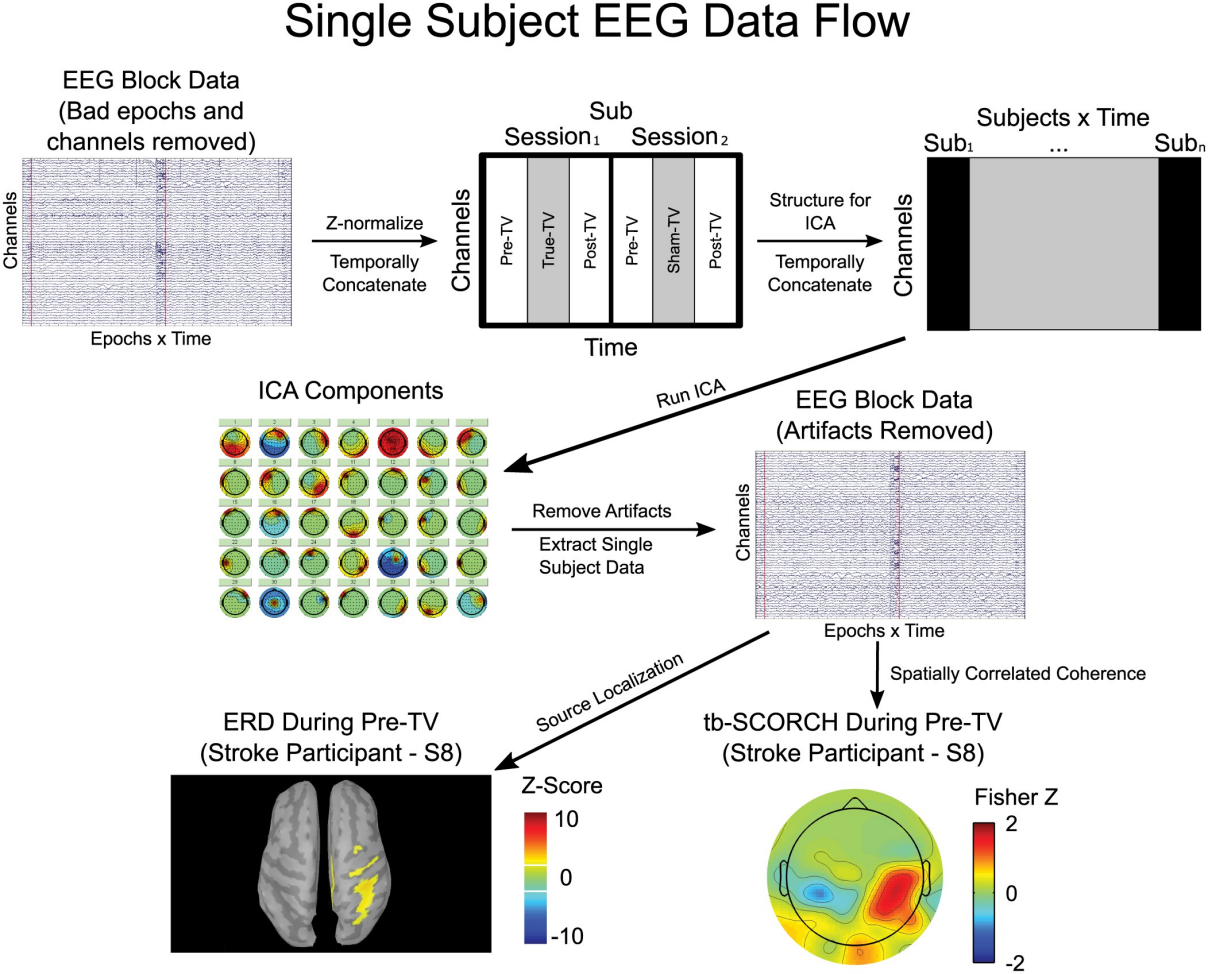
Single subject EEG data flow. Diagram of EEG data flow for artefact removal via ICA, and the tracking period beta band ERD and tb-SCORCH analyses for a single stroke participant (S8). After preprocessing, EEG data was standardized (z-score) and temporally concatenated across block, session, participant and group. The resulting matrix was then input into an ICA which output 64 independent temporal components. ICA components containing artefacts (eye blink, EMG and movement) were removed and the remaining components were transformed back to the EEG channel space where individual data were extracted. The tracking period beta band ERD averaged across epochs was thresholded at a z-score of two and tb-SCORCH topographic maps were displayed using the corresponding Fisher z-values. The hemisphere contralateral to the tested arm (paretic) was displayed on the left.

EEG data were post processed and analyzed using the EEGLAB toolbox (version v13.4.4b) [50] for storing and manipulating the data, FieldTrip (version 2016-01-03) [51] for removing bad trials and electrodes, Brainstorm (version 3.4) [52] for source localizing the data, and custom MATLAB scripts (version 2014a, MathWorks, Natick, Massachusetts). All EEG data were bandpass filtered (1-50Hz) using a fourth order zero-phase Butterworth filter. All EEG data were then epoched resulting in 96 epochs per block. EEG epochs were then baseline corrected (-0.5 to 0s) and bad channels and epochs were removed manually using FieldTrip’s visual inspection code (channel/epoch removed if variance/kurtosis >2 standard deviations from the mean, ft_rejectvisual, average number channels/epochs removed, 1.9/22.2). If a channel was rejected from the EEG data, its value was replaced with interpolated data from the surrounding electrodes [53]. EEG data were flipped so that the hemisphere contralateral to the arm being tested (paretic/non-dominant) was always represented on the left hemisphere. EEG data were separated into signal and artefactual components using an Adaptive Mixture Independent Component Analysis (AMICA) [54]. To ensure consistent artefact removal across blocks, sessions, participants and groups, EEG data were standardized (z-scored across electrode, time and epoch relative to baseline), temporally concatenated across independent variables (block, session, participant and group) for each electrode channel and used as input to the AMICA algorithm, Fig 2. This process resulted in 64 independent temporal components. Signal artefacts, including eye blink, EMG, and movement artefacts, were identified by distinct artefactual characteristics [55–58] and removed from the EEG data (25 components were removed). The remaining components were then transformed back to the EEG channel space where the individual block, session, participant and group data were extracted. Finally, EEG data were re-referenced to a common average reference for all data analyses excluding the functional connectivity analyses (below), which re-referenced the data to the average of the mastoids (Electrodes TP9 and TP10) [59]. Each re-reference technique reintroduced the FCz electrode to the data set.

#### 2.5.3. EEG activity

Source localization of EEG data was performed to examine the spatial characteristics of cortical activity. Distributed current dipole maps were computed in Brainstorm using the default MNI/Colin27 anatomical brain template [52]. The standard actiCAP electrode locations were fit to the scalp surface so that the Cz electrode location was at the vertex as described in the physiological measurements section. A boundary element model (BEM) was used to estimate of the forward model (OpenMEEG) [60, 61], and a depth-weighted minimum L2 norm estimator of cortical current density [62] was used to estimate the inverse model. The source localized data were then bandpass filtered (beta band: 13-26Hz) using a zero-phase fourth order Butterworth filter, squared to obtain power, averaged across epochs, normalized (see below), and averaged across the tracking period (0–7s). For cortical activation, a z-score normalization process was used to display the data shown in Fig 2 (baseline period: -0.5 to 0s). For statistical analyses and difference calculations, the power was normalized using percent change from baseline (baseline period: -0.5 to 0s),

$$\%\Delta \left(t\right)=100\times \frac{X\left(t\right)-baseline}{baseline}$$ (2)

Where %Δ(*t*) represents the percent change from baseline, ***X***(*t*) represents the power time series, *t* represents time, and *baseline* represents the average power in the baseline period. Beta band z-score and percent change from baseline values were characterized in terms of the associated event related desynchronization, ERD. ERD is a decrease in power relative to baseline, is thought to represent active cortical areas [49] and is defined here in terms of the average beta band event-related desynchronization during the tracking period.

ERD from the EEG source localization data was obtained from a region of interest (ROI) corresponding to the region of deficit activity in the stroke group. The deficit ROI was identified by comparing ERD within the Pre-TV blocks of the stroke-Vibe and stroke-Sham sessions with the control-Vibe and control-Sham sessions using a two-sample t-test, resulting in four unique maps of t-values that were subsequently thresholded (t ≥ 2.262). Vertices on the cortical surface that survived the threshold in all four maps were identified as the deficit ROI. The deficit ROI was located above the lateral pre-motor, motor and sensory cortices in the hemisphere associated with arm (paretic/non-dominant) movement. The mean ERD for the deficit ROI was then compared across blocks, sessions and groups. Correlations between the cortical activity (average of stroke-Vibe and stroke-Sham ERD in the Pre-TV block) of stroke participants and their functional ability (upper extremity motor FMA) were evaluated by calculating the correlation coefficient at each vertex on the cortical surface. Vertices on the cortical surface that resulted in significant correlations (t-test of correlation coefficient different than 0, p ≤ 0.05) were defined as the functional ROI. The functional ROI for ERD was located in parts of the paracentral, precuneus and superior parietal gyri in the hemisphere associated with arm (paretic/non-dominant) movement. The mean ERD in the deficit ROI and functional ROI for the Pre-TV block (averaged across stroke-Vibe and stroke-Sham) were also plotted against the upper extremity motor FMA for stroke participants.

#### 2.5.4. EEG connectivity

To examine EEG connectivity, all-to-all (connectivity between all possible pairs of EEG electrodes) temporal connectivity profiles were generated using magnitude squared coherence (*Coh*^2^) between electrodes *X* and *Y*,

$$Coh^{2}\left(f\right)=\frac{{\left|C_{XY}{\left(f\right)}_{\;}\right|}^{2}}{C_{XX}\left(f\right)\cdot C_{YY}\left(f\right)}$$ (3)

where *C*_*XY*_ is the cross spectrum between electrodes *X* and *Y*, *C*_*XX*_ is the auto spectrum of electrode *X*, *C*_*YY*_ is the auto spectrum of electrode *Y*, and *f* denotes frequency. Every EEG epoch was divided into 30 nonoverlapping windows, each containing 0.25s of data (epochs were divided up in this fashion to remove a comparable baseline coherence from tracking period coherence). Coherence was then calculated within each window using the epochs as the measure of consistency. For each participant, block, session and group, this resulted in a connectivity matrix that was 4225 (65x65 electrodes) by 30 elements for each frequency. The resulting connectivity matrices were then averaged across the beta band (13–26 Hz range), the connectivity during baseline was removed by masking the first 2 time points (representing the 0.5s of baseline data before the tracking period) and the remaining connectivity measures averaged across the last 28 time points (tracking period) to calculate the tracking period beta band task-based coherence (tb-Coh, matrix size: 65x65) [63]. A new method of quantifying tb-Coh (described below) is being introduced in this study. We extracted hemispheric and single electrode connectivity information from the connectivity matrix as ground truth for this new metric. Hemispheric tb-Coh was defined as tb-Coh between analogous electrodes in the two hemispheres. Hemispheric coherence values for electrodes along the midline were calculated as the average tb-Coh between the midline electrode and the electrodes to the immediate left and right. Finally, single-electrode tb-Coh was also extracted from the connectivity matrix and represented the tb-Coh of a single electrode with every other electrode.

To quantify spatial patterns of tb-Coh, we developed a spatially correlated coherence (SCORCH) metric. SCORCH was developed to describe and compare entire connectivity maps associated with a specific electrode rather than individual tb-Coh connections. Specifically, SCORCH quantified how well a participant’s single-electrode connectivity map matched a ground truth connectivity map (see following). The first step in calculating SCORCH was to generate a ground truth data set. Our ground truth data set was calculated by averaging the control-Vibe and control-Sham Pre-TV block tb-Coh matrices (matrix size: 65x65). We then spatially correlated each single-electrode tb-Coh map (65x1 array for each electrode) from every participant, block, group and session with the respective single-electrode tb-Coh map from the ground truth coherence matrix defined across controls. Correlation values for tb-SCORCH were Fisher z-transformed to normalize the population distribution for statistical testing. This resulted in a task-based SCORCH array (tb-SCORCH, 65x1) with a single correlation coefficient value for each single-electrode tb-Coh map in every participant, block, group and session. An electrode displaying a high value of tb-SCORCH indicated that its global coherence topography (i.e. network functional connectivity pattern) resembled that seen in the ground truth data set whereas a low value of tb-SCORCH implied the opposite.

Tb-SCORCH data were obtained from an electrode exemplifying the connectivity deficit in the stroke group. The deficit electrode was selected by comparing tb-SCORCH of the stroke-Vibe and stroke-Sham sessions with the control-Vibe and control-Sham sessions within the Pre-TV block using a two-sample t-test, resulting in four unique maps of t-values that were subsequently thresholded (t ≥ 2.262). If more than one electrode survived the threshold in all four maps, the electrode showing the largest reduction in tb-SCORCH in the stroke group was deemed the deficit electrode. The deficit electrode, identified as C3, was located above the sensorimotor cortices located in the hemisphere associated with arm (paretic/non-dominant) movement. The tb-SCORCH for the deficit electrode was compared across blocks, sessions and groups. To visualize any effects of TV, the tb-SCORCH in TV and Post-TV blocks from the control and stroke groups were compared to control Pre-TV block using a paired-sample t-test and two-sample t-test respectively with a false discovery rate (FDR) of a = 0.05 for multiple comparisons correction. Correlations between cortical connectivity (average of stroke-Vibe and stroke-Sham tb-SCORCH in the Pre-TV block) for the stroke participants and motor impairment (upper extremity motor FMA) were evaluated by calculating the correlation coefficient at every electrode. The electrode that resulted in a significant correlation (t-test of correlation coefficient different than 0, p < 0.05) and had the highest functional correlation was identified as the functional electrode. The functional electrode, identified as Cz, was centrally located between the motor cortices. The tb-SCORCH in the deficit and functional electrodes for the Pre-TV block (averaged across stroke-Vibe and stroke-Sham) were plotted against the upper extremity motor FMA for the stroke participants.

#### 2.5.5. Statistical analysis

Statistical analysis was performed using a combination of SPSS (version 25, IBM, Armonk, New York) and custom MATLAB scripts (version 2014a, MathWorks, Natick, Massachusetts). Changes in hand absolute error, speed, SD of speed, total path length, number of sub-movements, deficit ROI ERD and deficit electrode tb-SCORCH data were characterized across participants using three-way mixed ANOVAs of 3 (block: Pre-TV, TV, Post-TV) x 2 (session: Vibe, Sham) x 2 (group: Stroke, Control) with block and session as within-participant factors and group as the between-participant factor in the analysis. Appropriate two-way (mixed/repeated measure) ANOVAs, one-way repeated measure ANOVAs and t-tests were applied *post hoc* to characterize specific interaction effects identified in the 3-way mixed ANOVAs. If Mauchly’s Test of Sphericity indicated that the assumption of sphericity was violated, a Greenhouse-Geisser correction was used for the ANOVA tests. The Holm-Sidak method for correcting for multiple comparisons was used at each level (between multiple ANOVAs and t-tests) in the analysis except for multiple pairwise comparisons, where the Tukey *post hoc* test was applied. Raw p-values were reported and stated as significant if they survived the correction for multiple comparisons. A non-parametric bootstrap approach similar to the Zhou and Wong method [64] with 10000 iterations was used to generate the statistical distributions for the Tukey *post hoc* test. Statistical tests were performed with a Type I error rate of α = 0.05. Hand absolute error data was found to be positively skewed and was transformed to a normal distribution using a base 10 logarithmic function. Prior to statistical analysis of each variable, an outlier check was performed within groups (Stroke, Control) during blocks (Pre-TV, TV, Post-TV) and sessions (Vibe, Sham). If a participant’s data was deemed an outlier (exceeded 3 standard deviations of the group mean), then the participant’s data was excluded for that analysis, but might still be included in other analyses if not an outlier for the associated variables. Please see S1 Text for details surrounding our *post hoc* analysis examining changes in visuomotor performance during the Sham condition (specifically SD of hand speed).

## 3. Results

### 3.1. Movement kinematics

Analysis of the movement kinematics during the tracking period revealed differences between the control and stroke groups and improvements over time or blocks, Table 2. The three-way ANOVAs revealed a main effect of group for hand absolute error (F(1,18) = 30.75, p<0.001, η_p_^2^ = 0.63), SD of hand speed (F(1,18) = 15.46, p = 0.001, η_p_^2^ = 0.46), and number of sub-movements (F(1,18) = 17.06, p = 0.001, η_p_^2^ = 0.49). Hand absolute error (control: 0.81±0.17 cm; stroke: 2.43±1.36 cm) and SD of hand speed (control: 2.06±0.44 cm/s; stroke: 3.48±1.05 cm/s) were significantly higher in the stroke group when compared to controls, while the number of sub-movements during the tracking period was significantly lower in the stroke group (10.26±0.59) when compared to controls (11.37±0.61). The three-way ANOVAs also revealed a main effect of block for hand speed (F(1.15,20.75) = 6.75, p = 0.014, η_p_^2^ = 0.27), SD of hand speed (F(1.52,27.37) = 22.17, p<0.001, η_p_^2^ = 0.55), and total path length (F(1.15,20.75) = 6.75, p = 0.014, η_p_^2^ = 0.27). *Post hoc* analyses (Tukey test) of block differences revealed hand speed (Pre-TV: 6.95±0.56 cm/s; TV: 6.77±0.48 cm/s; Post-TV: 6.80±0.49 cm/s), SD of hand speed (Pre-TV: 2.94±1.13 cm/s; TV: 2.71±1.04 cm/s; Post-TV: 2.66±1.06 cm/s) and total path length (Pre-TV: 48.66±3.92 cm; TV: 47.41±3.36 cm; Post-TV: 47.60±3.45 cm) were significantly lower in the TV (q(38≥4.69, p≤0.005) and Post-TV (q(38)≥3.97, p≤0.021) block when compared to the Pre-TV block while the TV and Post-TV blocks showed similar activity (q(38) ≤1.68, p≥0.47). No other factors or interactions reached significance in the three-way ANOVAs of the movement kinematic variables (absolute error: p≥0.12; hand speed: p≥0.16; SD of hand speed: p≥0.46; total path length: p≥0.16; number of sub-movements: p≥0.13). Please see S1 Text for a supplementary statistical *post hoc* analysis of visuomotor performance data (SD of hand speed).

**Table 2:** Visuomotor performance data.

	Vibe Session			Sham Session		
	Pre-TV	TV	Post-TV	Pre-TV	TV	Post-TV
**Absolute Error (cm)**						
Control	**0.84** (0.25)	**0.80** (0.22)	**0.79** (0.20)	**0.84** (0.15)	**0.80** (0.17)	**0.77** (0.17)
Stroke	**2.39** (1.34)	**2.30** (1.42)	**2.49** (1.78)	**2.34** (1.13)	**2.56** (1.49)	**2.51** (1.34)
**Speed (cm/s)**						
Control	**6.84** (0.26)	**6.70** (0.17)	**6.75** (0.15)	**6.72** (0.13)	**6.67** (0.14)	**6.71** (0.13)
Stroke	**7.14** (0.98)	**6.88** (0.62)	**6.93** (0.68)	**7.11** (0.98)	**6.84** (0.62)	**6.82** (0.68)
**SD of Speed (cm/s)**						
Control	**2.27** (0.70)	**2.11** (0.58)	**1.99** (0.54)	**2.18** (0.34)	**1.94** (0.37)	**1.88** (0.38)
Stroke	**3.67** (1.22)	**3.39** (0.99)	**3.38** (0.98)	**3.65** (1.13)	**3.41** (1.07)	**3.39** (1.06)
**Total Path Length (cm)**						
Control	**47.86** (1.80)	**46.92** (1.18)	**47.26** (1.06)	**47.05** (0.88)	**46.65** (0.95)	**46.95** (0.89)
Stroke	**49.96** (6.89)	**48.16** (4.31)	**48.47** (4.76)	**49.76** (4.94)	**47.90** (5.63)	**47.71** (5.57)
**Sub-movements (#)**						
Control	**11.14** (0.72)	**11.42** (0.69)	**11.29** (0.68)	**11.40** (0.57)	**11.50** (0.72)	**11.46** (0.67)
Stroke	**10.24** (0.60)	**10.27** (0.68)	**10.22** (0.68)	**10.32** (0.69)	**10.30** (0.58)	**10.23** (0.62)

Tracking period visuomotor performance data (hand absolute error, hand speed, SD of hand speed, total path length of the hand and number of hand sub-movements) during the vibration (Vibe) and sham (Sham) session. Data were averaged across participants with the standard deviation given in parentheses.

### 3.2. Initial tracking ERD

**Fig 3:**
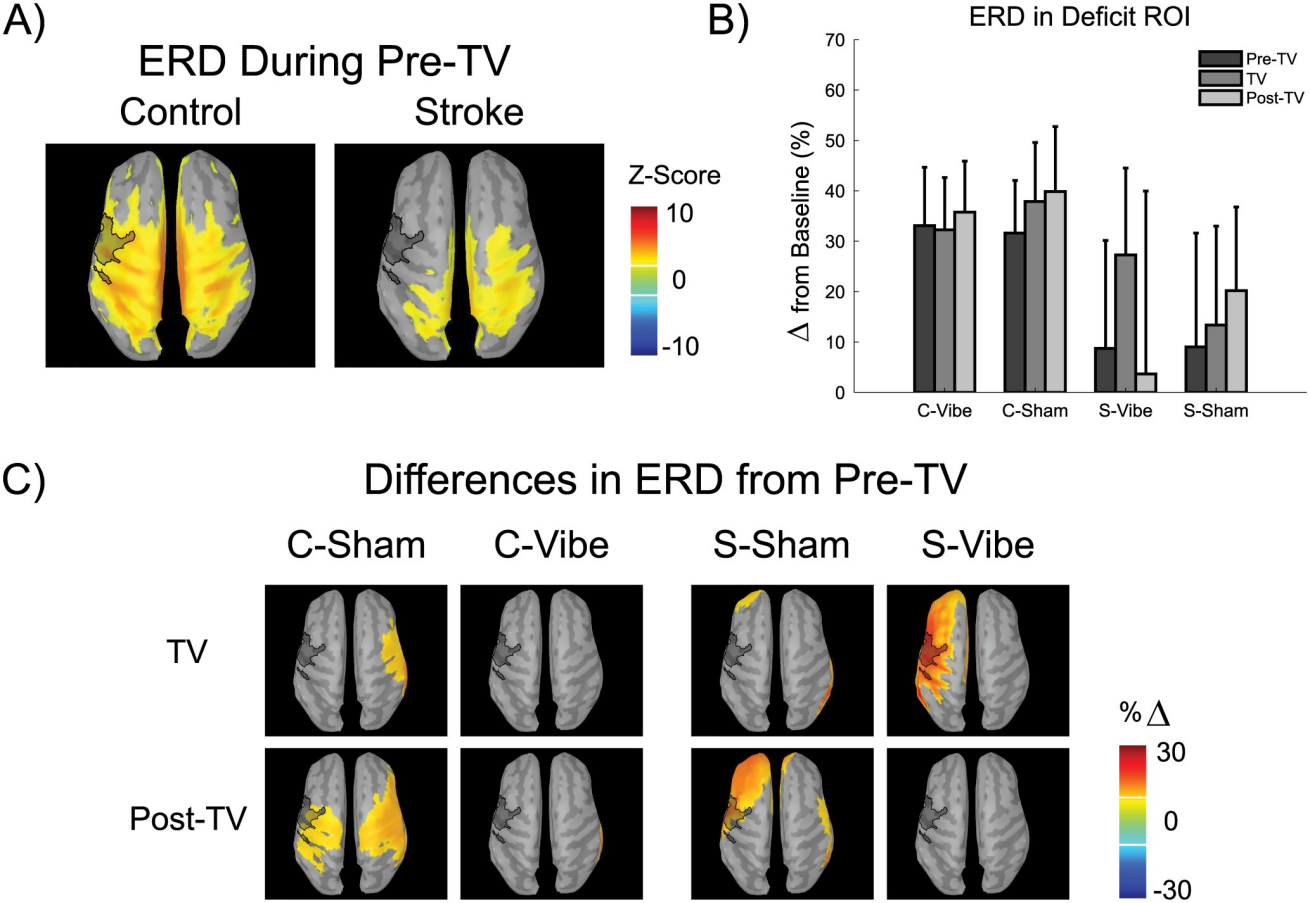
EEG source localization of beta band ERD during the tracking period. The hemisphere contralateral to the tested arm (paretic/non-dominant) is displayed on the left. A) Average ERD during the Pre-TV block. Z-scores averaged across participants and sessions are shown for each group. Only values above or below a z-score threshold of ±2 are displayed. Positive values indicate ERD while negative values indicate a resynchronization, relative to baseline. The dark translucent overlay denotes the deficit ROI. B) Average ERD in the deficit ROI expressed as the percent change from baseline averaged across participants (error bars denote the 95% confidence interval about the mean). C) Difference ERDs from Pre-TV (Control: C-Vibe and C-Sham, Stroke: S-Vibe and S-Sham). The percent change (%Δ) values denotes the difference between the respective block and the Pre-TV block for each session with a positive/negative %Δ indicating a larger/smaller ERD within the respective block. Only values above or below a %Δ difference of ± 9 are displayed for clarity. The dark translucent overlay denotes the deficit ROI.

ERD was examined during the Pre-TV block to identify any initial differences in the movement related activity across the cortex between the control and stroke groups, Fig 3A. In the control group, ERD was identified in premotor, motor, sensory and parietal cortices and was located bilaterally. The stroke group showed ERD in the premotor, motor, sensory and parietal cortices that was lateralized to the hemisphere ipsilateral to the paretic limb, with some ERD in the parietal cortex of the contralateral hemisphere. Along with the drastic decrease in spatial extent of cortical activation in the stroke group, the areas that did display ERD were lower in magnitude when compared to controls.

### 3.3. Deficit ROI ERD

The deficit ROI was located above the lateral pre-motor, motor and sensory cortices in the hemisphere associated with arm (paretic/non-dominant) movement, Fig 3A and 3C. Stroke participant S9’s deficit ROI ERD data was excluded from this analysis because their data was found to be an outlier (although S9 data was retained in other analyses). ERD in the deficit ROI was similar across blocks and sessions but different between groups, Fig 3B. ERD in the deficit ROI was significantly lower in the stroke group (13.71±23.66%Δ) when compared to the control group (35.08±14.93%Δ) (F(1,17) = 5.67, p = 0.029, η_p_^2^ = 0.25, 3-Way ANOVA). No other factors or interactions reached significance (p≥0.061). Fig 3B shows the ERD in the deficit ROI; during the sham session, both the control (Pre-TV: 31.62±14.61%Δ; TV: 37.88±16.37%Δ; Post-TV: 39.86±18.04%Δ) and stroke (Pre-TV: 9.03±29.37%Δ; TV: 13.36±25.54%Δ; Post-TV: 20.19±21.61%Δ) groups’ deficit ROI ERD increased over time (blocks). The application of tendon vibration increased the ERD in the deficit ROI for the stroke group (Pre-TV: 8.73±27.88%Δ; TV: 27.29±22.40%Δ) to levels near those of the control group (C Pre-TV: 33.08±16.22%Δ), but the increase in ERD did not persist in the Post-TV block (3.66±47.24%Δ). Tendon vibration did not alter the ERD in the control group (TV: 32.27±14.49%Δ), although, it did appear to prevent the increase in ERD over time (blocks) seen in the control and stroke sham sessions.

### 3.4. Differences in spatial ERD

Spatial maps of the differences between TV/Post-TV and Pre-TV ERD were examined to investigate whether the trends found in the deficit ROI were present more generally in the movement related activity across the cortex (Fig 3C). An increase in ERD over time (blocks) in the control-Sham session was present bilaterally in the premotor, motor, sensory and parietal cortices and grew in magnitude over time (see Fig 3C). Application of tendon vibration in the control group caused minimal changes in ERD across the cortex but prevented the increases in ERD that were seen over time in the sham session. The increase in ERD over time in the stroke-Sham session was less pervasive than in the control-Sham session and was focused in the lateral premotor and frontal cortices contralateral to paretic arm movement (Fig 3C). When tendon vibration was applied to stroke participants, the ERD increased in the lateral frontal, premotor, motor and sensory cortices contralateral to paretic arm movement. The increase in ERD with tendon vibration in the stroke group did not persist in the Post-TV block and prevented the lateralized increases in ERD seen over time in the sham session.

### 3.5. Initial tracking task-based coherence (tb-Coh)

**Fig 4:**
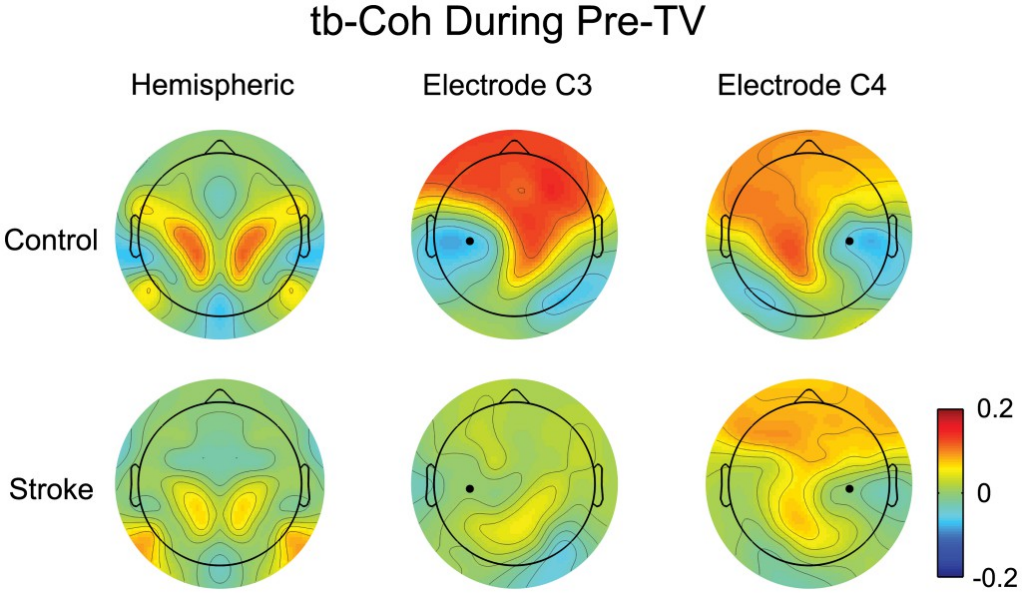
Tracking period task-based coherence (tb-Coh) in the beta band during Pre-TV. The hemisphere contralateral to the tested arm (paretic/non-dominant) is displayed on the left. The spatial variation in tb-Coh (coherence change from baseline period) averaged across participants and sessions is shown for each coherence measure. Values of tb-Coh were interpolated between electrodes. Negative values indicate a decrease in tb-Coh while positive values indicate an increase in tb-Coh relative to the baseline period. The black dot on the single electrode coherence maps indicates the location of the electrode.

Electrode level tb-Coh was examined during the Pre-TV block to instill confidence in our tb-Coh analysis prior to introducing tb-SCORCH. Maps for hemispheric, electrode C3 (electrode over the sensorimotor cortex contralateral to the movement arm) and electrode C4 (electrode over the sensorimotor ipsilateral to the movement arm) tb-Coh are shown for the control and stroke groups in Fig 4. In general, the control and stroke groups had similar patterns of tb-Coh for each metric with what appeared to be higher levels of tb-Coh in the control group compared to the stroke group. Hemispheric tb-Coh indicated strong levels of connectivity between the homologous electrodes located above the premotor, motor, sensory and partial areas. In control participants, the tb-Coh for electrode C3 had relatively high values of tb-Coh above the contralateral motor and sensory cortices extending up into the frontal areas of both hemispheres with relatively low values of tb-Coh located around electrode C3, suggesting the importance of frontal/sensorimotor communication during a figure-8 tracking task. Stroke participant tb-Coh for electrode C3 showed minimal changes from baseline with small increases occurring above the ipsilateral parietal cortex and contralateral motor and sensory cortices. The tb-Coh for electrode C4 was comparable between control and stroke groups and was a mirror image of electrode C3’s tb-Coh map in controls, although with slightly smaller connectivity.

### 3.6. Initial tracking tb-SCORCH

**Fig 5:**
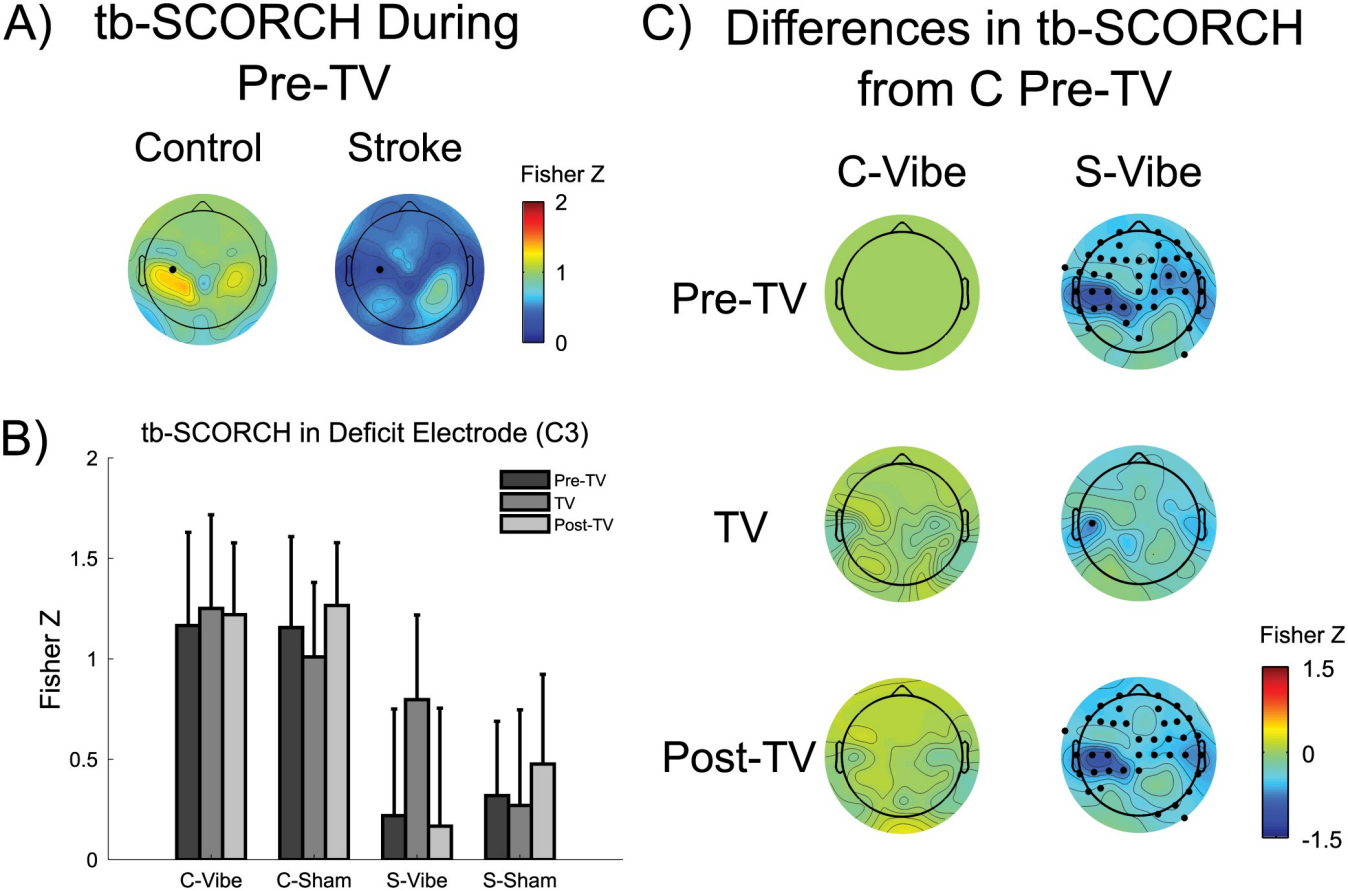
Beta band tb-SCORCH during the tracking period. The hemisphere contralateral to the tested arm (paretic/non-dominant) is displayed on the left. A) tb-SCORCH in the Pre-TV block (Control: C-Vibe and C-Sham, Stroke: S-Vibe and S-Sham). The tb-SCORCH Fisher z-values averaged across participants and sessions are shown for each group. Larger Fisher z-values indicate a stronger correlation of connectivity patterns between the group and the ground truth connectivity pattern (average of control-Vibe and control-Sham Pre-TV) during tracking. The black dot indicates the deficit electrode (C3). Values of tb-SCORCH were interpolated between electrodes B) tb-SCORCH in the deficit electrode. The bar chart shows tb-SCORCH Fisher z-values averaged across participants. Error bars denote the 95% confidence interval about the mean. C) Differences in beta band tb-SCORCH from Pre-TV for control participants. Black dots indicate the electrodes that were significantly different, using an FDR correction at α = 0.05. The Fisher z-values correspond to the differences between the respective block and the control Pre-TV block with a positive/negative Fisher z-value indicating an increase/decrease in the correlation of the connectivity maps within the respective block. Values of tb-SCORCH were interpolated between electrodes.

The tb-SCORCH was examined during the Pre-TV block to identify differences in the global functional connectivity patterns between control and stroke groups (Fig 5A). Two nodes with high levels of tb-SCORCH in the control group were identified bilaterally above motor, sensory and parietal areas. The node above the contralateral hemisphere (associated with arm movement) was spatially larger and contained higher tb-SCORCH values than the node in the ipsilateral hemisphere. The tb-SCORCH pattern was similar in the stroke group but contained considerably lower tb-SCORCH values across the brain. The stroke group had the highest values of tb-SCORCH in the node above the ipsilateral hemisphere while the node in the contralateral hemisphere was almost nonexistent.

### 3.7. Deficit electrode tb-SCORCH

The deficit electrode, identified as C3, was located above the sensorimotor cortices located in the hemisphere associated with arm (paretic/non-dominant) movement (Fig 5A). The tb-SCORCH of the deficit electrode was significantly lower in the stroke group (0.37±0.54) compared to controls (1.18±0.50) (F(1,18) = 11.74, p = 0.003, η_p_^2^ = 0.39, 3-Way ANOVA). The three-way mixed ANOVA of the deficit electrode’s tb-SCORCH also revealed a significant interaction between block and group (F(2,36) = 3.42, p = 0.044, η_p_^2^ = 0.16) and between block and session (F(2,36) = 5.57, p = 0.008, η_p_^2^ = 0.24). No other factors or interactions in the three-way ANOVA reached significance (p≥0.25).

When examining the block by group interaction, the *post hoc analysis* (1-Way ANOVA) for blocks revealed no significant results for the control (Pre-TV: 1.16±0.59; TV: 1.13±0.53; Post-TV: 1.24±0.44) (F(1.28,11.50) = 0.99, p = 0.36, η^2^ = 0.10) or stroke groups (Pre-TV: 0.27±0.55; TV: 0.53±0.50; Post-TV: 0.32±0.67) (F(2,18) = 3.26, p = 0.062, η^2^ = 0.27) although there was a trend towards the TV block having a higher value in the stroke participants. The *post hoc analysis* (two-sample t-test) for groups indicated a significantly larger tb-SCORCH in the controls compared to the stroke group for each block (t(18)≥2.59, p≤0.019).

When examining the block by session interaction, the *post hoc* analysis (1-Way ANOVA) of blocks revealed significant results for the vibration session (F(1.51,28.60) = 4.91, p = 0.022, η^2^ = 0.21) with a significantly lower tb-SCORCH in the Pre-TV (0.69±0.83) and Post-TV (0.69±0.85) blocks compared to the TV (1.02±0.65) block (q(38)≥3.843, p≤0.029) but no difference between the Pre-TV and Post-TV blocks (q(38) = 0.009, p~1). No significant results for the Sham session were found (Pre-TV: 0.74±0.71; TV: 0.64±0.69; Post-TV: 0.87±0.66) (F(2,38) = 2.35, p = 0.109, η^2^ = 0.11; 1-Way ANOVA). The *post hoc* analysis (paired-sample t-test) for sessions indicated a significantly higher tb-SCORCH in the vibration session when compared to the sham session for the TV block (t(19) = 2.65, p≤0.016) and no differences between sessions for the Pre-TV or Post-TV blocks (t(19)≤1.71, p≥0.1).

Fig 5B displays the tb-SCORCH in the deficit electrode and shows that the significant interactions of block/group and block/session found in the three-way ANOVA were most likely driven by the stroke group’s response to tendon vibration. The application of tendon vibration increased the amount of deficit electrode tb-SCORCH in the stroke group (Pre-TV: 0.22±0.74; TV: 0.80±0.59) closer to the level of tb-SCORCH in the controls (Pre-TV: 1.17±0.65), but this increase in tb-SCORCH did not persist in the stroke Post-TV block (0.16±0.82). Tendon vibration only slightly increased the tb-SCORCH in the control group (TV: 1.25±0.65). Finally, there was not an increase in tb-SCORCH over time (blocks) during the control-Sham session (Pre-TV: 1.16±0.63; TV: 1.01±0.52; Post-TV: 1.27±0.44) or stroke-Sham session (Pre-TV: 0.32±0.52; TV: 0.27±0.67; Post-TV: 0.48±0.62) as was seen in the ERD of the sham session.

### 3.8. Differences in spatial tb-SCORCH

The differences in tb-SCORCH between TV/Post-TV and Pre-TV were examined at every electrode to see if the results found in the deficit electrode were present in the global functional connectivity patterns across the cortex (Fig 5C). Application of the tendon vibration in the controls caused minimal changes in tb-SCORCH across the cortex. Contrary to controls, when tendon vibration was applied in stroke participants, an increase in tb-SCORCH was found throughout the brain, nearly eradicating the large deficit in tb-SCORCH located above the sensorimotor areas in the hemisphere associated with arm movement. The increase in tb-SCORCH with application of tendon vibration in the stroke participants did not persist in the Post-TV block (see Fig 5C).

### 3.9. Deficit electrode tb-Coh

**Fig 6:**
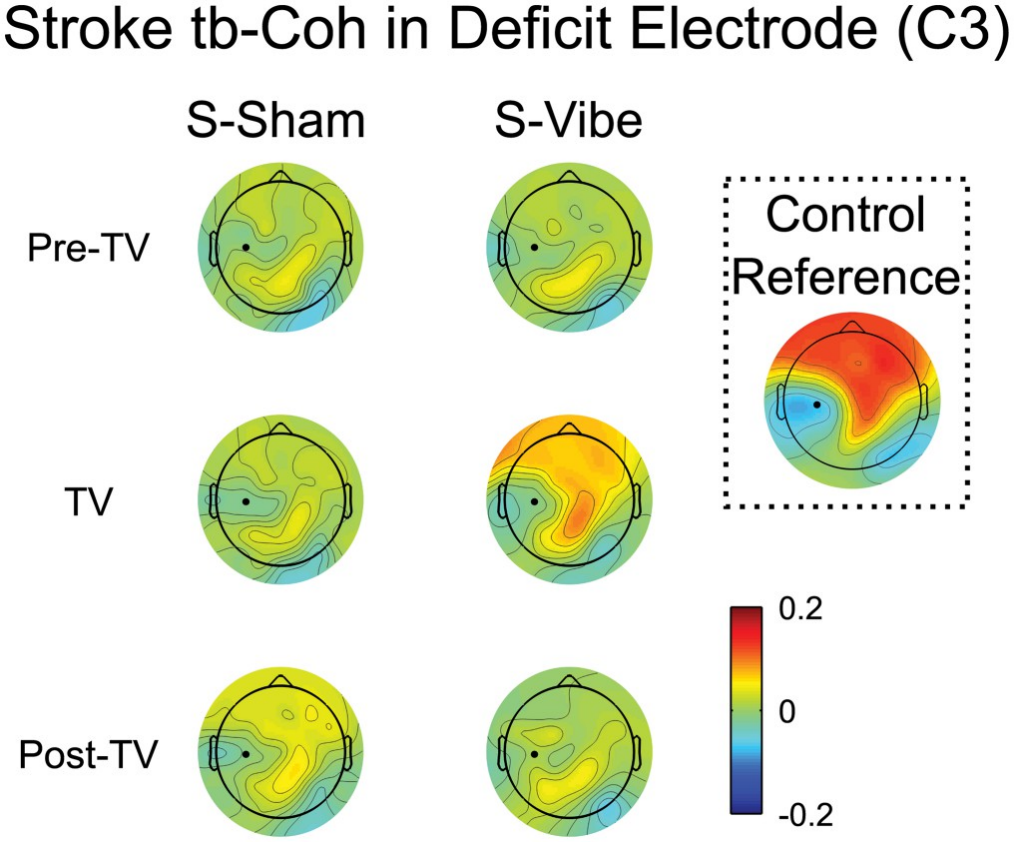
Stroke beta band tb-Coh in the deficit electrode (C3) during the tracking period. The hemisphere contralateral to the tested arm (paretic/non-dominant) is displayed on the left. The tb-Coh (coherence change from baseline period) averaged across participants is shown for the deficit electrode (C3). The control reference inset shows electrode C3’s tb-Coh averaged across participants and sessions (control-Vibe and control-Sham) for the Pre-TV block. Values of tb-Coh were interpolated between electrodes for mapping. Positive/negative values indicate an increase/decrease in tb-Coh relative to the baseline period. The black dots indicate the location of the deficit electrode (C3).

While tb-SCORCH identifies electrodes with global connectivity pattern differences, it does not indicate how those patterns look and/or change. To visualize the underlying connectivity pattern changes associated with the significant results from the tb-SCORCH analysis, the deficit electrode (C3) tb-Coh was examined during the Pre-TV, TV and Post-TV blocks for the stroke-Vibe and stroke-Sham sessions and visually compared to the control Pre-TV block (Fig 6). As noted previously, the deficit electrode’s tb-Coh for the stroke group displayed minimal changes from baseline connectivity with small increases occurring above the ipsilateral parietal cortex and contralateral motor and sensory cortices for all blocks except TV. During the TV block, the deficit electrode’s tb-Coh resembled the control group (see inset of Fig 6); both displayed high values of tb-Coh above the contralateral motor and sensory cortices extending up into the frontal areas of both hemispheres with low values of tb-Coh located around the deficit electrode.

### 3.10. ERD and tb-SCORCH correlations

**Fig 7:**
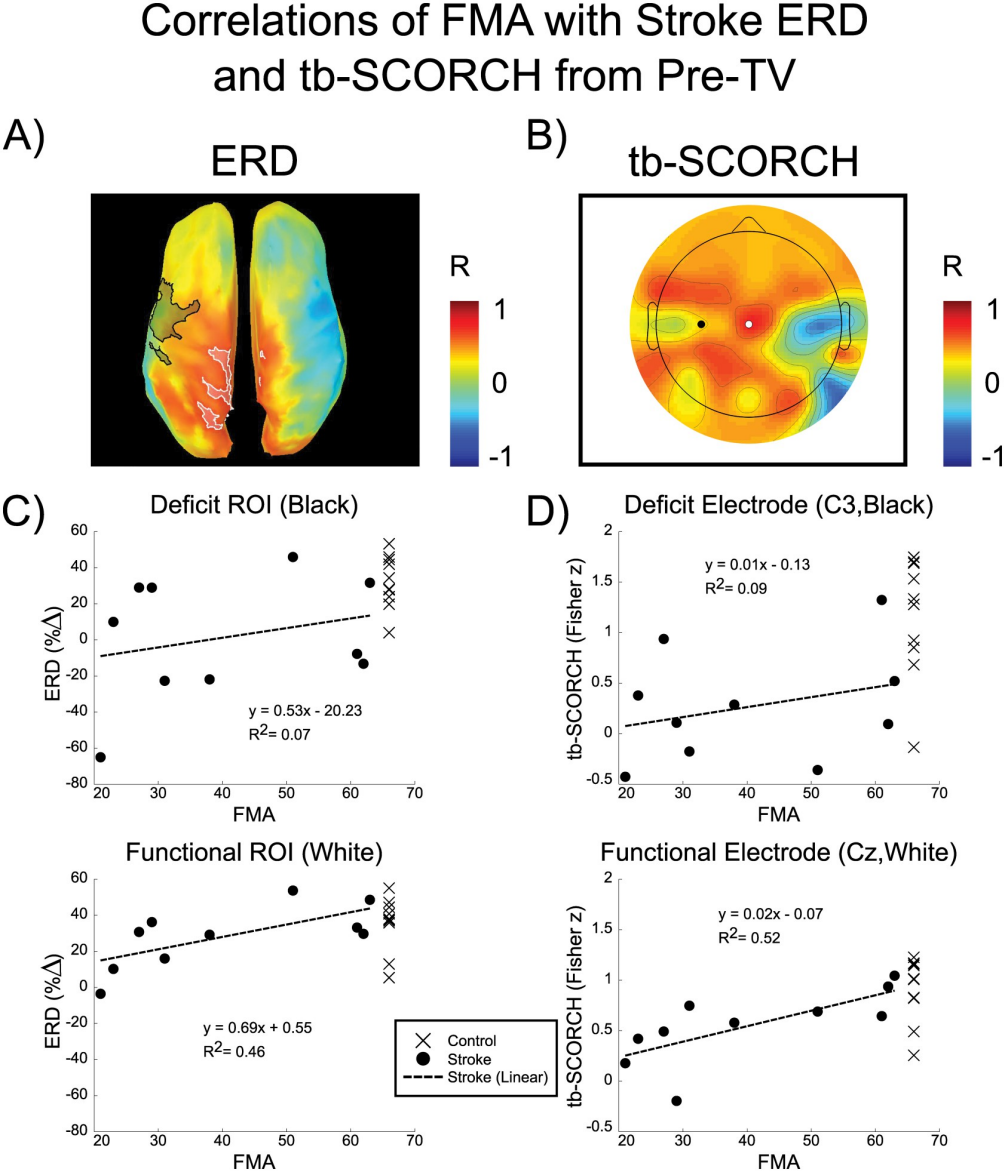
Correlations of FMA with stroke ERD and tb-SCORCH from Pre-TV. Correlations of upper extremity motor FMA scores with tracking period beta band ERD and tb-SCORCH during the Pre-TV block for stroke participants. The hemisphere contralateral to the tested arm (paretic/non-dominant) is displayed on the left. A) Correlations of vertex-wise ERD within the Pre-TV block (averaged across sessions: stroke-Vibe and stroke-Sham) with upper extremity motor FMA scores. Black and white shaded overlays indicate the deficit and functional ROIs, respectively. B) Correlations of tb-SCORCH for each electrode, within the Pre-TV block (averaged across sessions: stroke-Vibe and stroke-Sham), with upper extremity motor FMA scores. Correlation values are interpolated between electrodes for display purposes. The black and white dots indicate the deficit (C3) and functional (Cz) electrodes, respectively. C) Correlation of the ERD within the Pre-TV block for the deficit and functional ROIs with upper extremity motor FMA scores. ERD was averaged across sessions (stroke-Vibe and stroke-Sham) before correlation with the motor FMA. Control ERD during the Pre-TV block for the same ROIs was averaged across sessions (control-Vibe and control-Sham) and plotted against a perfect upper extremity motor FMA of 66. D) Correlation of stroke electrode tb-SCORCH within the Pre-TV block with upper extremity motor FMA scores. Tb-SCORCH was averaged across sessions (stroke-Vibe and stroke-Sham) before correlation with the motor FMA. Control tb-SCORCH during the Pre-TV block for the same electrodes were averaged across sessions (control-Vibe and control-Sham) and plotted against a perfect upper extremity motor FMA of 66.

Deficit ROI ERD (cortical activity) and deficit electrode (C3) tb-SCORCH (cortical connectivity) were correlated with upper extremity motor FMA (function ability) to determine whether the level of cortical deficit predicted functional outcome in the stroke participants. Fig 7A and 7B show the whole brain correlations of ERD and tb-SCORCH with upper extremity motor FMA. Both images display similar patterns of correlation with the largest positive values of correlation occurring over the sensorimotor and parietal areas associated with paretic arm movement and the largest negative correlation values occurring over the sensorimotor areas associated the non-paretic limb movement. The correlation of function with activity and connectivity of the deficit ROI/electrode did not have a linear relationship, with values of R^2^ = 0.07 (p = 0.45) and R^2^ = 0.09 (p = 0.40), respectively (Fig 7C and 7D). However, the functional ROI for ERD was correlated with functional outcome in parts of the paracentral, precuneus and superior parietal gyri in the hemisphere associated with arm (paretic/non-dominant) movement (R^2^ = 0.46, p = 0.03), while for tb-SCORCH the functional electrode (Cz), which is centrally located between motor cortices, was best correlated with functional outcome (R^2^ = 0.52, p = 0.02), (Fig 7C and 7D).

## 4. Discussion

**Fig 8:**
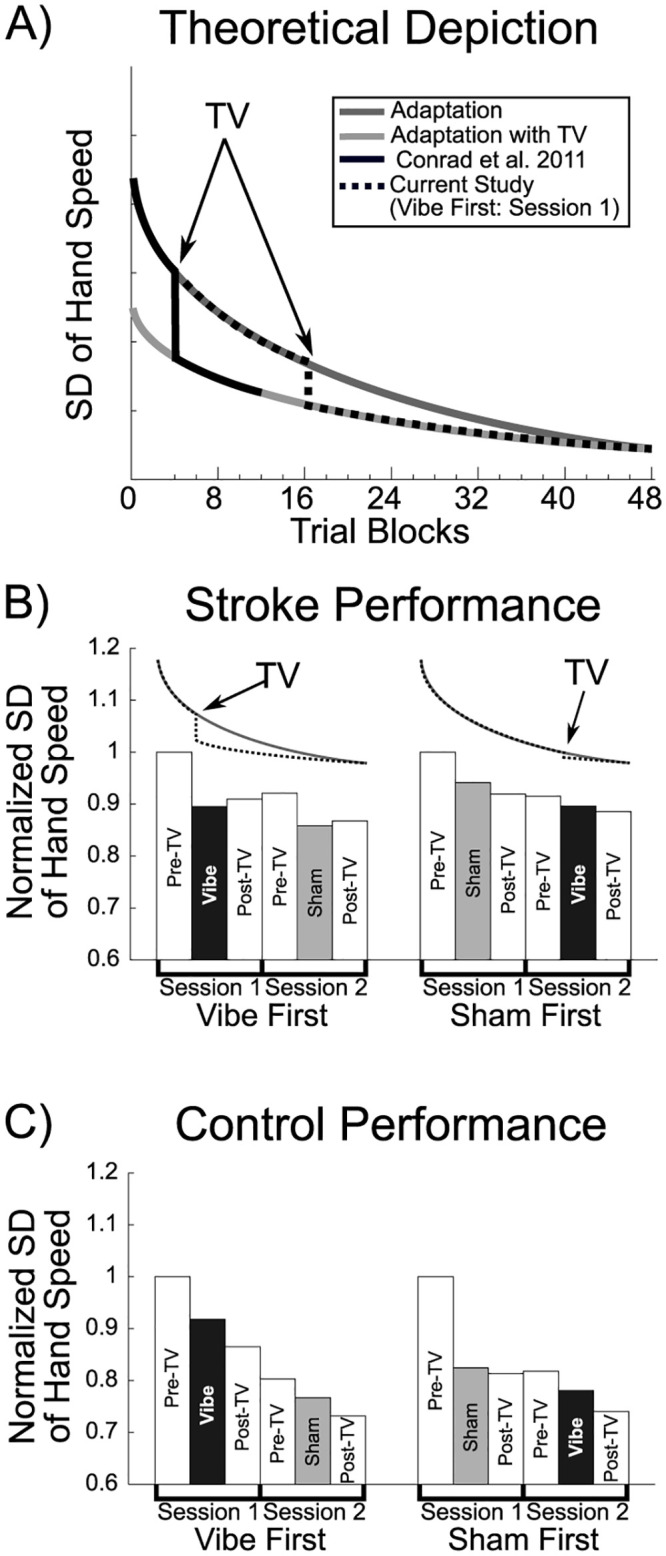
Tracking performance. Tracking period SD of hand speed adaptation over time with possible step increase in adaptation due to application of tendon vibration. A) Theoretical depiction of typical tracking period SD of hand speed adaptation curve and hypothesized TV adaptation curve. When TV is applied near the beginning of the adaptation process [14] there would theoretically be a larger decrease in SD of hand speed than when TV is applied later (current study). B) Stroke SD of hand speed during tracking period. Data were averaged across the 5 stroke participants that received TV during session 1 (Vibe First) and the across the 5 stroke participants that received the sham TV during session 1 (Sham First). The curves above the bar plots display the theoretical tracking period SD of hand speed adaptation curve with the point at which TV was applied. C) Control SD of hand speed during tracking period. Data were averaged across the 5 control participants that received TV during session 1 (Vibe First) and the across the 5 control participants that received the sham TV during session 1 (Sham First). Data for B and C were normalized (for each participant) by calculating the ratio of each condition relative to session 1’s Pre-TV condition. Error bars were withheld for display purposes.

In this study, we set out to identify changes in cortical activity and connectivity associated with tendon vibration during visuomotor tracking in people with stroke. We tested the hypothesis that wrist flexor tendon vibration increases cortical activity (ERD) and connectivity (tb-Coh and tb-SOCRCH) of the cortex in people with stroke. The results demonstrated stroke-related deficits in cortical activity and functional connectivity during a figure-8 tracking task when compared to controls, with the largest deficits localized to the sensorimotor cortices associated with the paretic arm. The extended testing protocol needed to measure EEG produced results consistent with motor adaptation in the sham condition, with evidence for an increase in cortical activity in stroke and controls over the test period. When tendon vibration was applied in stroke participants, sensorimotor cortical activity and connectivity contralateral to the paretic arm increased to levels near those of control participants. The presence of increased cortical activity and functional connectivity in people with stroke suggests that tendon vibration during arm movement might improve cortical function.

### 4.1. Visuomotor performance

Visuomotor tracking performance (SD of hand speed) improved for both Vibe and Sham over the extended test procedures in the current study. Specifically, we noted that kinematic variables were not statistically different across the vibration and sham experiments using our current study design. A significant improvement in tracking performance is observed with the application of tendon vibration when a shorter experimental timeframe is used [14]. However, the experimental protocol of the current study was extended to increase the number of trials for EEG data processing. It appeared that the repeated visuomotor tracking task might have produced gradual improvements in performance through practice effects. The later timing of vibration along a theoretical motor learning curve, which follows a power law, may have contributed to the lower levels of improvement in tracking performance in the current study, Fig 8A [65–67].

A proposed framework describing interactions of a theoretical motor learning curve and timing of vibration on kinematic parameters is shown in Fig 8A, and the data from Table 2 are shown graphically in Fig 8B and 8C for SD of hand speed. Note that for stroke participants, when tendon vibration was applied early in the theoretical motor learning process as in the Conrad and colleagues study [14], tendon vibration would be expected to produce a large, statistically detectable improvement in tracking performance. While an improvement in tracking performance was observable in the stroke participants that received tendon vibration during the first session of the current study, tendon vibration was applied much later along the theoretical motor learning curve than in the prior study [14] resulting in a smaller expected improvement in tracking performance. When the stroke participants that received tendon vibration during session 2 were introduced to tendon vibration, it is possible that the participants’ tracking performance was very close to the theoretical performance floor, leaving little room for a tendon vibration effect.

Interestingly, for control participants tendon vibration seemed to disrupt the typical power law curve associated with motor learning [65–67] and reduced tracking performance improvements. Similar to stroke participants, the time that tendon vibration was applied during the theoretical motor learning process seemed to impact the magnitude of the vibration effect in controls. Improvements in tracking performance were disrupted when vibration was applied in session 1, but largely unaffected if applied in session 2, as a majority of motor learning had most likely already occurred, Fig 8C. Please see S1 Text for further discussion of our *post hoc* analysis examining the effects of vibration timing on visuomotor tracking performance (SD of hand speed).

### 4.2. Increase in ERD over time

The increases in ERD over time in both the control and stroke groups during sham testing is consistent with motor learning. Previous studies examining cortical oscillations during motor tasks have reported increases in alpha and beta band desynchronization (cortical activity) over the sensorimotor cortices during and after learning [68–72]. Further, cortical activity and size of excitable cortex increases up to the point that a motor task is explicitly learned, after which the activity and size of excitable cortex returns to, or below, baseline levels [70, 73]. The absence of an ERD return to baseline in the current study suggests that acquisition of explicit knowledge of the tracking task was inhibited, possibly due to varying the start direction of the target (clockwise or counterclockwise) on a trial by trial basis.

During the vibration experiments, the increase in ERD over time was not seen in either the control or stroke groups, suggesting that tendon vibration affected the motor learning process. One explanation is that vibration facilitated motor learning and allowed the tracking task to be explicitly learned, which would cause the ERD to return to baseline levels [70, 73]. Applying tDCS over the motor cortex facilitates learning in a serial reaction time task, suggesting that additional input to the cortex facilitates the learning process [74]. Motor learning also improves after a vibration sensory attention task, indicating increased learning rates can be obtained by selective modulation of proprioceptive input [13]. While the ERD did return to baseline levels after the application of tendon vibration, the control and stroke groups had differing ERD responses as well as opposite behavioral responses with tendon vibration; this indicates that the ERD return to baseline was probably not associated with acquisition of explicit knowledge (at least in controls) and suggests that tendon vibration might be altering cortical activity via a different process.

### 4.3. Effect of vibration on cortical function

The application of tendon vibration during the figure-8 tracking task had different effects on the control and stroke participants suggesting that the somatosensory signal was processed differently in each group. The lack of cortical activity/connectivity change in the control group could result from the brain correctly interpreting the tendon vibration as noise (i.e. task-irrelevant). Cortical networks can facilitate relevant sensory information while inhibiting unrelated sensory inputs [75–79]. Increases in EEG band power (reduced ERD magnitude in our case) have been linked to sensory suppression of somatic stimuli [80]. A consequence of increased sensory noise via tendon vibration during the figure-8 tracking task could be poorer behavioral performance [81–85] which was observed in the control group. In a similar way, the increased cortical activity/connectivity observed in the stroke group in response to tendon vibration could reflect an inability to gate task-irrelevant sensory information [75–78]. Although people with stroke have deficits gating sensory stimuli [86], behavioral performance was not disrupted by tendon vibration in the current study. In fact, tendon vibration normalized stroke cortical activity/connectivity and improved behavioral performance.

Sensory loss in the stroke population, Table 1, can significantly affect upper limb movements given that sensory feedback (proprioception) is a requirement for fine motor control [18]. Inadequate integration of proprioception may cause the cortical network responsible for controlling the paretic limb to function improperly and possibly even become more reliant on visual feedback, ultimately resulting in poor motor control in the paretic arm [87]. The flow of additional somatosensory information via tendon vibration, which mimics peripheral system inputs during movements [18], may help to boost task-relevant somatosensory signals of the limb through stochastic resonance [88], and help the system overcome the sensory deficits typically seen in people with stroke [46]. Increased cortical activity in the deficit ROI (Fig 3B) and to a lesser extent, throughout the lesioned hemisphere (Fig 3C) supports this interpretation and is consistent with other studies examining the effects of vibration on the feet, fingers and arm that have shown similar increases in cortical activity [25, 89, 90]. Further, increased functional connectivity in the deficit electrode (Fig 5B) and throughout the brain (Fig 5C) suggests wrist flexor tendon vibration during a figure-8 tracking task has the capability of normalizing widespread cortical networks. This result supports previous findings showing external stimulation (e.g. TMS, tDCS) can elicit changes in cortical connectivity distant from the stimulation site and improve cortical connectivity in stroke patients [26–28, 91].

While the data suggest that the flow of additional somatosensory information via tendon vibration affects sensorimotor control, tendon vibration may have also affected task-awareness. It is likely the participants noticed the tendon vibrator was off during the Sham session and on during the Vibe session, increasing task-awareness. Decreased awareness (poorer performance and response times) is related to decreased beta band power in visual tasks [92, 93]. Although there were beta band power fluctuations in the visuomotor tracking task, stroke participants demonstrated a decrease in beta band power (increase in ERD) during tendon vibration that was associated with improved performance (Figs 3 and 8). Further, increased awareness relates to improved performance in control and diseased populations [92, 93], whereas tendon vibration appeared to improve performance in the stroke group and disrupt performance in the control group (Fig 8B and 8C).

### 4.4. Cortical areas correlated with impairment

Whole brain cortical activity and connectivity were positively correlated with upper extremity motor FMA scores in the lesioned hemisphere and negatively correlated with FMA in the non-lesioned hemisphere. These trends suggest that outcomes of stroke improve as the lesioned/non-lesioned hemisphere becomes more/less active or functionally connected, suggesting more symmetric cortical activity/connectivity patterns result in higher functional outcomes. Shifts from asymmetrical to symmetrical hemispheric cortical organization with training are associated with clinical improvements [94, 95].

Interestingly, cortical activity/connectivity in medial regions of the cortex was most strongly associated with impairment after stroke. The paracentral, posterior cingulate, precuneus and superior parietal gyri are higher order association areas and have been associated with a variety of functions including visuo-spatial imagery, episodic memory, self-processing, consciousness, attention, visuo-motor integration, audio-visual integration and motor control [96–102]. These areas are highly connected and constitute a central hub in the brain’s integrative pathways [103]. Integration areas are likely to be affected after stroke, regardless of lesion location due to their underlying connectedness and the connectedness after stroke appears to play a critical role in recovery of function [104–106].

While the cortical areas correlated with impairment are interesting, Fig 7C and 7D revealed that 2 control participants had low ERD and tb-SCORCH values in the functional ROI and electrode while not being impaired, breaking the trend of high cortical measures corresponding to high functional ability. To better understand the relationship between the functional regions and impairment level, the correlation of functional ROI ERD (cortical activity) and functional electrode (Cz) tb-SCORCH (cortical connectivity) with upper extremity motor FMA (function ability) were recomputed with the inclusion of control participant data. The functional ROI (activity) correlation was changed to R^2^ = 0.25 (p = 0.03) from R^2^ = 0.46 (p = 0.03) and the functional electrode (connectivity) correlation was changed to R^2^ = 0.46 (p = 0.001) from R^2^ = 0.52 (p = 0.02). Although including the control data decreased the p-values of the correlation test, the R^2^ values also decreased. The drop in R^2^ values may be due to the functional areas being defined using only stroke participant data, suggesting these are the regions functionally meaningful in stroke participants but not necessarily functionally related to the task in controls. This may seem counterintuitive, but after stroke, cortical reorganization can occur [26, 95, 107]. Additionally, the upper extremity motor FMA is designed to assess impairment after stroke and is an inadequate measure for discerning differences of functional ability in neurologically-intact participants [47]. Future studies, where correlation of control data is needed, should include other functional measures that can differentiate control participants such as the Box and Block test [108].

### 4.5. Study limitations

The present study’s experimental design controlled for several confounding factors, such as motor learning, ordering effects, placebo effects, fatigue and consistent artefact removal. However, we allowed participants to sustain their normal daily routines and did not restrict the use of external stimulants such as nicotine and caffeine before sessions. While these stimulants may have altered EEG signals, many nicotine and caffeine users may have an ongoing dependence to the substances whereby recent stimulant abstinence still results in abnormal EEG activity [109, 110] and might even be a larger disrupter of EEG activity [111]. In addition to stimulant usage, other experiment-related factors may have impacted the observed changes in beta band activity and connectivity including fatigue [112], EEG contamination by muscle activity and exclusion of true EEG signals. While participants were given breaks throughout the experiment to minimize fatigue, a few of the lower functioning stroke participants reported being tired during the experiments. Despite this, the stroke group’s movement kinematics still improved during the stroke-Sham and stroke-Vibe sessions, indicating that fatigue played a minimal role. Although the AMICA algorithm was used to remove artefactual EEG signals, it is possible that the algorithm did not fully separate signals and artefacts; this may have resulted in the subsequent EEG source imaging analyses missing some cortical signal and/or containing some artefactual components.

There are possible limitations to the functional connectivity analyses, which are impacted by volume conduction and reference electrode choice. Volume conduction results in the spatial blurring of cortical point sources measured at the scalp and can produce significant coherence between EEG electrodes even if a true connectivity does not exist [113]. Techniques have been developed to mitigate this issue including imaginary coherence [114], orthogonalization techniques [115, 116], and task-based coherence [117]. In the present study, we used task-based coherence [117] which removes volume conduction and baseline coherence from the task period coherence [118]. Although task-based coherence results in near zero connectivity values for adjacent electrodes, we minimized this issue by comparing the same connections across tasks rather than different connections within tasks. Coherence is also impacted by the choice of reference electrode or referencing scheme (common average, linked mastoids, etc.) [59, 119, 120]. Using a single electrode reference during coherence analyses can inflate or deflate values depending on how active the reference site is (larger values at the reference site are detrimental to coherence) [121]. To mitigate this issue, we referenced our EEG data to the average of the mastoids to better approximate an ideal, zero-potential reference [59].

Lastly, examining the short-term effects of tendon vibration in chronic stroke participants may have failed to illuminate the full repertoire of tendon vibration benefits during arm movements. While the present results suggest that tendon vibration improves cortical activity/connectivity and motor learning rate in chronic stroke participants, a single session of tendon vibration did not seem to alter motor performance level nor sustain cortical activity/connectivity improvements after removal. When long-term training studies apply pure sensory training or electrical stimulation before physical therapy, functional outcomes improve compared to physical therapy alone [122, 123]; this suggests that prolonged exposure to tendon vibration in conjunction with therapy may be necessary to sustain cortical improvements as well as generate overall performance increases in the chronic stroke participants. Unlike chronic stroke patients, the central nervous system of acute stroke patients has been recently damaged and is in the process of relearning and reorganizing [95, 107, 124–126]. Applying tendon vibration during training throughout this critical phase may help cortical networks relearn more normal patterns of activity/connectivity and potentially improve motor performance. To further examine the effect tendon vibration has on stroke motor learning, it would be interesting to extend our experiment by adding a fourth tracking block after the Post-TV blocks. If cortical activity stays the same or even decreases between the Post-TV block and the newly introduced fourth block, that would be evidence for tendon vibration increasing motor learning. If cortical activity were to increase in the fourth block relative to the Post-TV block, that would suggest that tendon vibration is disrupting a motor learning/consolidation process. A fifth block could also be introduced hours/days later to test the effect that tendon vibration has on motor memory formation. Examining movement kinematics in this block would indicate if tendon vibration altered motor memory formation.

## 5. Conclusions

EEG measures of brain activity and connectivity provided insight into motor learning and effects of vibration. The application of vibration to the wrist flexor tendons during hand tracking increased cortical activity and connectivity of the deficit regions in people with stroke. The increases in cortical activity and connectivity with vibration normalized patterns of activity. These findings suggest that reactivation of normal cortical networks via tendon vibration may be useful during physical rehabilitation of stroke patients; however, more research is needed to illuminate the interaction between motor learning and potential tendon vibration benefits during task performance in people with stroke.

## Supporting information

S1 Text Kinematic order effect. (DOCX)
