# Supplementary material for: Cortical effects of wrist tendon vibration during an arm tracking task in chronic stroke survivors: An EEG study
Source: PLoS One. 2023 Dec 21;18(12):e0266586. doi: 10.1371/journal.pone.0266586 (PMC10735026; doi:10.1371/journal.pone.0266586)
Supplement: S1 Text — (DOCX) [file pone.0266586.s001.docx]

Cortical effects of wrist tendon vibration during an arm tracking task in chronic stroke survivors: An EEG study

Dylan B. Snyder ^1¶^, Scott A. Beardsley ^1&^, Allison S. Hyngstrom ^2&^, Brian D. Schmit ^1&*^

^1#a^ Department of Biomedical Engineering, Marquette University and Medical College of Wisconsin, Milwaukee, Wisconsin. Olin Engineering Center Room 206, 1515 West Wisconsin Avenue, Milwaukee, WI 53233

^2#b^ Department of Physical Therapy, Marquette University, Milwaukee, Wisconsin. Schroeder Complex Room 346, 560 North 16^th^ Street, Milwaukee, WI 53233

^*^ Corresponding Author:

Email: brian.schmit@marquette.edu (BS)

Address: PO Box 1881, Milwaukee, WI 53201-1881

Phone: 414-288-6125

**^¶^**This author contributed to this work: conceptualization, data curation, formal analysis, investigation, methodology, project administration, software, validation, visualization, writing – original draft preparation, writing – review & editing.

^&^These authors also contributed equally to this work: conceptualization, funding acquisition, methodology, project administration, resources, supervision, writing – review & editing.

**S1 Text: Kinematic order effect**

**Methods**

For the core analyses of our study, we had 2 groups (stoke and control) each consisting of 10 participants. Every participant in each group came into our lab on 2 separate days to perform the vibratory session and the sham session. The sessions were counterbalanced across participants such that 5 participants performed the vibratory session on the 1^st^ day (sham session on the 2^nd^ day) and the other 5 participants performed the sham session on the 1^st^ day (vibratory session on the 2^nd^ day). Behavioral performance data in Table 2 (located in the main research article) show results of the 2 groups (stroke and control) over the 2 sessions (vibratory and sham).

As a *post hoc* analysis to determine why vibration did not result in a significant improvement in behavioral performance data, specifically SD of hand speed, we decided to break apart each group (stroke and control) into 2 subgroups of 5 participants each (those that performed the vibratory session first and those that performed the sham session first) to examine whether some sort of learning was masking the effect of vibration. SD of hand speed data in Table A and Fig 8 (located in the main research article) show that each of the 4 subgroups (Control Vibe First, Control Sham First, Stroke Vibe First, Stroke Sham First) completed both sessions (vibratory and sham) but the order that the sessions were completed was flipped (due to the counterbalancing that was done).

Changes in SD of speed were characterized across participants using a three-way mixed ANOVA of 3 (block: Pre-TV, TV, Post-TV) x 2 (session: Vibe, Sham) x 4 (subgroup: Control Vibe First, Control Sham First, Stroke Vibe First, Stroke Sham First) with block and session as within-participant factors and group as the between-participant factor in the analysis. Appropriate two-way (mixed/repeated measure) ANOVAs, one-way (normal/repeated measure) ANOVAs and t-tests were applied *post hoc* to characterize specific interaction effects identified in the 3-way mixed ANOVA. If Mauchly’s Test of Sphericity indicated that the assumption of sphericity was violated, a Greenhouse-Geisser correction was used for the ANOVA tests. The Holm-Sidak method for correcting for multiple comparisons was used at each level (between multiple ANOVAs and t-tests) in the analysis except for multiple pairwise comparisons, where the Tukey *post hoc* test was applied. Raw p-values were reported and stated as significant if they survived the correction for multiple comparisons. A non-parametric bootstrap approach similar to the Zhou and Wong method [64] with 10000 iterations was used to generate the statistical distributions for the Tukey *post hoc* test. Statistical tests were performed with a Type I error rate of α = 0.05.

**Results**

Analysis of SD of hand speed during the tracking period revealed differences between the groups, blocks and sessions (Table A). The three-way ANOVAs revealed a main effect of group (F(1,16)=5.4, p=0.009, η_p_^2^=0.50), a main effect of block (F(1.42,23.79)=22.11, p<0.001, η_p_^2^=0.58), an interaction between group and session (F(3,16)=9.50, p<0.001, η_p_^2^=0.64), and an interaction between group, block, and session (F(6,32)=3.56, p=0.008, η_p_^2^=0.40). No other factors or interactions in the three-way ANOVA reached significance (p≥0.30).

**Table A: SD of Hand Speed.**

|  |  | **Vibe Session** | | |  | **Sham Session** | | |
| --- | --- | --- | --- | --- | --- | --- | --- | --- |
|  |  | Pre-TV | TV | Post-TV |  | Pre-TV | TV | Post-TV |
| **Vibe First** |  |  |  |  |  |  |  |  |
| Control |  | **2.81** (0.56) | **2.56** (0.43) | **2.41** (0.39) |  | **2.23** (0.33) | **2.12** (0.37) | **2.03** (0.43) |
| Stroke |  | **3.92** (1.47) | **3.42** (1.05) | **3.48** (1.11) |  | **3.56** (1.22) | **3.29** (1.11) | **3.30** (1.01) |
| **Sham First** | |  |  |  |  |  |  |  |
| Control |  | **1.73** (0.21) | **1.66** (0.27) | **1.57** (0.25) |  | **2.13** (0.39) | **1.75** (0.27) | **1.73** (0.30) |
| Stroke |  | **3.41** (1.03) | **3.35** (1.05) | **3.28** (0.95) |  | **3.75** (1.17) | **3.53** (1.16) | **3.47** (1.23) |

Tracking period SD of hand speed (cm/s) during the vibration (Vibe) and sham (Sham) session. Control and stroke participant data were split into subgroups depending on whether the participants received TV during session 1 (Vibe First) or sham TV during session 1 (Sham First).

To examine the interaction between group, block, and session, two-way ANOVAs with block and session as within-participant factors were performed on each of the 4 subgroups (Control Vibe First, Control Sham First, Stroke Vibe First, Stroke Sham First). The *post hoc* analyses revealed a main effect of session for the Control Vibe First group (F(1,4)=10.85, p=0.03, η_p_^2^=0.73) and the Control Sham First group (F(1,4)=38.25, p=0.003, η_p_^2^=0.91) with the session performed first having a significantly higher SD of hand speed (Control Vibe First: Vibe [2.59±0.45 cm/s] Sham [2.13±0.37 cm/s]; Control Sham First: Vibe [1.65±0.23 cm/s] Sham [1.87±0.31 cm/s]). A main effect of block was also found for the Control Vibe First group (F(2,8)=8.84, p=0.009, η_p_^2^=0.69), Control Sham First group (F(2,8)=27.89, p<0.001, η_p_^2^=0.88), and Stroke Vibe First group (F(2,8)=6.44, p=0.022, η_p_^2^=0.62). No other factors or interactions in the two-way ANOVAs reached significance (p≥0.055), although the main effect of session did approach significance in the Stroke Sham First group (F(1,4)=7.18, p=0.055, η_p_^2^=0.64) and the interaction between block and session did approach significance in the Control Sham First group (F(2,8)=5.26, p=0.08, η_p_^2^=0.57).

Tukey *post hoc* results examining the significant main effect of block revealed significantly lower SD of hand speed in the Post-TV block compared to the Pre-TV block in the Control Vibe First (Pre-TV: 2.52±0.42 cm/s; TV: 2.34±0.36 cm/s; Post-TV: 2.22±0.44 cm/s) (q(8)=5.92, p=0.007), Control Sham First (Pre-TV: 1.93±0.29 cm/s; TV: 1.70±0.26 cm/s; Post-TV: 1.65±0.28 cm/s) (q(8)=8.05, p=0.001), and Stroke Vibe First groups (Pre-TV: 3.74±1.34 cm/s; TV: 3.36±1.06 cm/s; Post-TV: 3.39±1.05 cm/s) (q(8)=4.59, p=0.034). It also revealed significantly lower SD of hand speed in the TV block compared to the Pre-TV block for the Control Sham First (q(8)=9.95, p<0.001) and Stroke Vibe First groups (q(8)=4.17, p=0.049). No other significant results were found between blocks for the groups (p≥0.094) although there was a trend towards a lower SD of hand speed in the TV block compared to the Pre-TV block in the Control Vibe First group (q(8)=3.46, p=0.094).

**Discussion**

These results suggest that control participants can retain behavioral improvements in SD of hand speed over separate days while stroke participants struggle to do so. When examining Fig 8, it appears that stroke participants can also retain improvements over separate days. However, the lowered magnitude of improvement (stroke: ~15%; control: ~25%, Fig 8) and the increased variability of stroke participants compared to controls (Table A) seems to have reduced statistical power. Further, control participants show earlier (TV block as opposed to Post-TV block) behavioral improvements (lower SD of hand speed) within sessions when tendon vibration is applied on day 2 (Sham First). Contrarily, stroke participants only show within session behavioral improvements when tendon vibration is applied on day 1 (Vibe First). Fig 8 visually confirms these statistical findings by showing how the early application of tendon vibration (Vibe First, session 1) hinders the normal performance seen in controls (Sham First, session 1) while it improves the normal performance seen in stroke participants (Sham First, session 1). When tendon vibration is applied late (Sham First, session 2), it appears to have a much smaller effect on normal performance (Vibe, session 2) seen in the control and stroke groups.
